# Stokes drift and its discontents

**DOI:** 10.1098/rsta.2021.0032

**Published:** 2022-06-13

**Authors:** Jacques Vanneste, William R. Young

**Affiliations:** ^1^ School of Mathematics and Maxwell Institute for Mathematical Sciences, University of Edinburgh, Edinburgh EH9 3FD, UK; ^2^ Scripps Institution of Oceanography, University of California at San Diego, La Jolla, CA 92093-0213, USA

**Keywords:** Stokes drift, surface gravity waves, wave–mean flow interaction

## Abstract

The Stokes velocity $u^{\text{S}}$, defined approximately by Stokes (1847, *Trans. Camb. Philos. Soc.*, **8**, 441–455.), and exactly via the Generalized Lagrangian Mean, is divergent even in an incompressible fluid. We show that the Stokes velocity can be naturally decomposed into a solenoidal component, $u_{\text{sol}}^{\text{S}}$, and a remainder that is small for waves with slowly varying amplitudes. We further show that $u_{\text{sol}}^{\text{S}}$ arises as the sole Stokes velocity when the Lagrangian mean flow is suitably redefined to ensure its exact incompressibility. The construction is an application of Soward & Roberts’s glm theory (2010, *J. Fluid Mech.*, **661**, 45–72. (doi:10.1017/S0022112010002867)) which we specialize to surface gravity waves and implement effectively using a Lie series expansion. We further show that the corresponding Lagrangian-mean momentum equation is formally identical to the Craik–Leibovich (CL) equation with $u_{\text{sol}}^{\text{S}}$ replacing $u^{\text{S}}$, and we discuss the form of the Stokes pumping associated with both $u^{\text{S}}$ and $u_{\text{sol}}^{\text{S}}$.

This article is part of the theme issue ‘Mathematical problems in physical fluid dynamics (part 1)’.

## 1. Introduction

Surface gravity waves induce a rectified motion of fluid particles and thus a wave-averaged difference between the mean Eulerian velocity, $u^{\text{E}}$, and the mean Lagrangian velocity $u^{\text{L}}$ [1,2]

1.1 $$u^{L}=u^{E}+u^{\text{S}}.$$

Above, $u^{\text{S}}$ is the Stokes velocity, also known as Stokes drift. The Stokes velocity is fundamental to understanding wave-averaged effects, such as the CL vortex force and the Stokes–Coriolis force, in the wave-averaged momentum and vorticity equations [3–7].

The Stokes velocity can be defined exactly at finite wave amplitude using Generalized Lagrangian Mean (GLM) theory [8,9]. This exact GLM $u^{\text{S}}$ is rotational and compressible, even if the underlying fluid motion is irrotational and incompressible [10]. Expansion in powers of a wave-amplitude parameter ϵ produces the standard approximation [2,11] to the Stokes velocity

1.2 $$u^{\text{S}}=\bar{\left(\xi_{1}\;\cdot \;\nabla \right)\;u_{1}}.$$

The overbar in (1.2) denotes a running time mean, or phase average. In (1.2), $u_{1}$ is the linear (first order in ϵ) velocity of the wave and the associated displacement $\xi_{1}$ is defined by $\partial_{t}\xi_{1}=u_{1}$ and $\bar{\xi_{1}}=0$. (The subscript 1 indicates the first-order fields throughout.) The small-amplitude approximation to $u^{\text{S}}$ in (1.2) is also rotational and compressible: assuming only that $\nabla \;\cdot \;\xi_{1}=0$, McIntyre [10] shows from (1.2) that

1.3 $$\nabla \;\cdot \;u^{\text{S}}=\partial_{t}{\left(\frac{1}{2}\bar{\xi_{1i}\xi_{1j}}\right)}_{,ij}.$$

The time derivative of an averaged quadratic quantity in (1.3) entails the same slow-modulation assumption that underlies the concept of group velocity and so introduces a second small parameter, μ. The Eulerian mean velocity, $u^{\text{E}}$ in (1.1), is incompressible and thus the divergent $u^{\text{S}}$ in (1.3) implies a divergent Lagrangian mean velocity.

**Figure 1.**
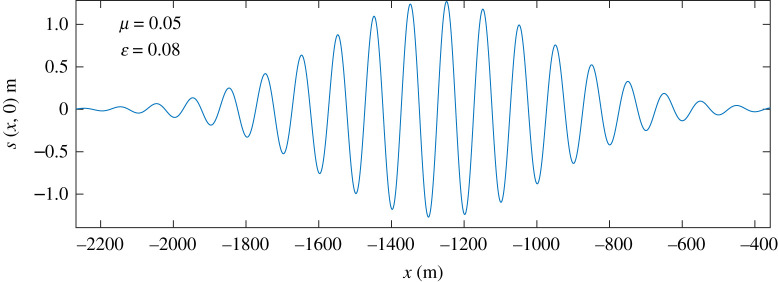
The sea-surface displacement, s(x,t), of a packet of surface gravity waves at t=0. The envelope is Gaussian, $a\exp[-{\left(x-x_{0}-\frac{1}{2}ct\right)}^{2}/2\ell^{2}]$, and the carrier wavenumber is k=2π/100 m. The 100 m wave length corresponds to an 8 s period and a group velocity c/2 of $6.24\;m\;s^{-1}$. The maximum surface displacement a=1.27 m corresponds to maximum wave orbital speed $1\;m\;s^{-1}$. The modulation parameter is μ=1/(kℓ)=0.05 and the wave slope is ϵ=ka=0.08. (Online version in colour.)

**Figure 2.**
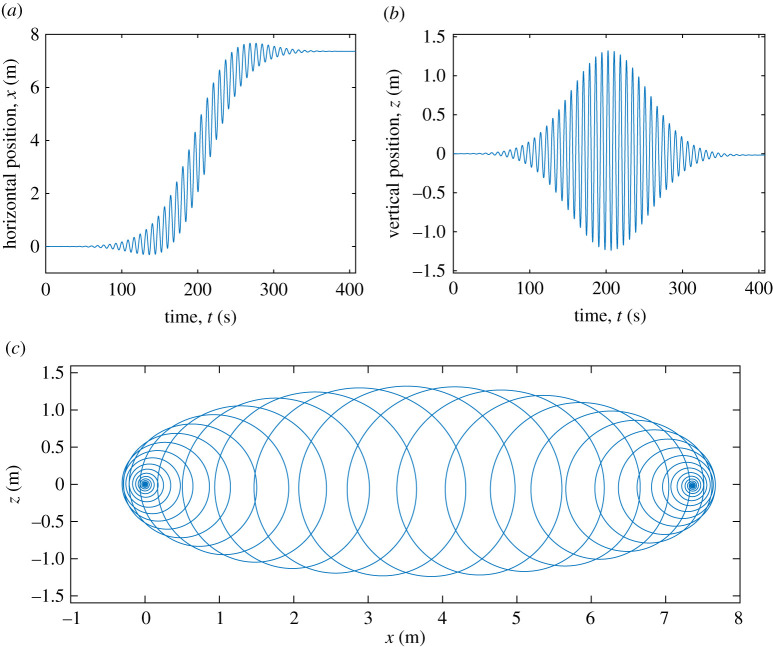
Trajectory of a fluid particle in the linear velocity field of the wave packet in figure 1. Panels (a,b) show the x- and z-displacements as functions of time; panel (c) shows the trajectory. In this computation, we assume that the depth d is much greater than the packet length scale ℓ so that the second-order Eulerian mean flow is negligible in the wave-active zone. (Online version in colour.)

In figure 1, we illustrate the role of the two small parameters ϵ and μ, by considering a weakly nonlinear, slowly modulated two-dimensional packet of deep-water surface gravity waves. The Stokes expansion [1,10] is justified by the weakly nonlinear assumption that the wave slope is small: ϵ=ak≪1, where k is the wavenumber and a is the amplitude of the surface displacement. In this example the slow-modulation parameter is $\mu ={\left(k\ell \right)}^{-1}\ll 1$ where ℓ is length scale of the packet envelope. Figure 2 shows the motion of a fluid particle in the velocity field of this wave.

Despite (1.3), and the exact results provided by GLM, some authors are reluctant to accept the reality of non-zero $\nabla \;\cdot \;u^{\text{S}}$. Moreover, discontent with $\nabla \;\cdot \;u^{\text{S}}\neq 0$ is sometimes confounded with unease over the vertical component of the Stokes velocity, $w^{\text{S}}=\hat{z}\;\cdot \;u^{\text{S}}$. For example, rather than taking the vertical component of (1.2), McWilliams *et al.* [12] defines a ‘vertical Stokes pseudo-velocity’ which, together with the horizontal components of $u^{\text{S}}$, makes an incompressible three-dimensional ‘Stokes pseudo-velocity’.

Mellor [13], while emphasizing that $u^{\text{S}}$ is divergent, is unwilling to accept a non-zero vertical component $w^{\text{S}}$: ‘a mean vertical drift is not acceptable’. In the view of concerns with the mean vertical drift $w^{\text{S}}$ it is reassuring that particle tracking velocimetry can be used to observe vertical Lagrangian displacements, $w^{\text{S}}\neq 0$, beneath groups of deep-water waves [14,15]: the mean vertical drift is upward as a wave packet arrives and downwards as the packet departs. (For the wave packet in figure 1, the maximum vertical displacement resulting from $w^{\text{S}}$ is about 4.8 cm—this is not visible in figure 2.) After the passage of the packet a fluid particle returns to its initial depth. It is a *net vertical displacement* that is unacceptable: transient vertical motion, on time scales longer than a 10-s wave period, and shorter than the 100-s packet transit time, is not a concern.

Mellor critiques the Craik & Leibovich ([3,4]) vortex-force formulation of wave-mean interaction by arguing that CL and subsequent authors incorrectly assume that the divergence of $u^{\text{S}}$ is zero. A different interpretation is that CL and many followers assume that the wave field has no temporal modulation so that the right of (1.3) is conveniently zero. For example, McWilliams & Restrepo [7] claim to prove $\nabla \;\cdot \;u^{\text{S}}=0$. But examination of this argument shows that [7] assumes that there is no temporal modulation of the wave field. This raises the issue of whether the CL formulation is incomplete or misleading in situations with temporal modulation of the wave field, e.g. in ocean observations [16] and in modelling the growth of swell [17].

In this paper, we revisit the concept of Stokes velocity. For surface gravity waves, we exhibit a natural Helmholtz decomposition of $u^{\text{S}}$ in (1.2) and argue that the solenoidal component, $u_{\text{sol}}^{\text{S}}$, can advantageously replace $u^{\text{S}}$ in most situations. We emphasize that the familiar form (1.2) of the Stokes velocity is not unique but depends on a specific definition of the Lagrangian-mean flow, namely the GLM definition of Andrews & McIntyre [8]. An alternative definition, proposed by Soward & Roberts [18] and closely related to classical averaging and its Lie series implementation (e.g. [19,20]), leads to a solenoidal Lagrangian-mean velocity with $u_{\text{sol}}^{\text{S}}$ as the corresponding Stokes velocity. This alternative definition, known as ‘glm’ but better characterized as ‘solenoidal Lagrangian mean’, has the added benefit of coordinate independence in any geometry, unlike standard GLM (see [21] for other coordinate-independent definitions of the Lagrangian mean). We show that, for surface gravity waves, the associated Lagrangian-mean momentum equation governing the dynamics of the Eulerian mean flow is the CL equation with $u_{\text{sol}}^{\text{S}}$ replacing $u^{\text{S}}$.

The difference between GLM and glm is vividly illustrated with an example proposed by O. Bühler (personal communication, 2021). Consider a bucket of initially motionless water. If the water is agitated, for example, by pressure forcing at the surface, then the potential energy of the water is increased, or equivalently the centre of mass of the water is elevated above its initial height. The fluid at the bottom of Bühler’s bucket, however, cannot move in the vertical and so any definition of ‘Lagrangian mean’ that tracks the position of the centre of mass of the fluid—such as GLM—will be divergent in this situation, even though the velocity of the water is entirely incompressible. Conversely, any definition of ‘Lagrangian mean’ resulting in a strictly solenoidal Lagrangian mean velocity—such as glm—cannot track the centre of mass of the fluid.

The plan of the paper is as follows. In §2, we sketch the derivation of the standard form (1.2) of the Stokes velocity, give its Helmholtz decomposition, and show how a simple modification of this derivation, implementing an alternative Lagrangian-mean flow definition, naturally brings about the velocity $u_{\text{sol}}^{\text{S}}$. In §3, we examine the respective role of $u^{\text{S}}$ and $u_{\text{sol}}^{\text{S}}$ in Stokes pumping, which is the mechanism whereby the horizontal divergence of the Stokes transport drives an Eulerian mean flow. In §4, we show how the glm approach enables the systematic construction of solenoidal Lagrangian-mean and Stokes velocities up to arbitrary algebraic accuracy in ϵ. We explain how Lie series provide both an interpretation and an efficient implementation of this construction, and we derive the glm version of the CL equations. Section 5 gives the conclusion.

## 2. The Stokes velocity $u^{S}$ and its solenoidal part $u_{\mathrm{sol}}^{S}$

### (a) Derivation of the Stokes velocity

We start by recalling the traditional derivation of the Stokes velocity in (1.2). The position x(t) of a fluid particle is determined by solving

2.1 $$\frac{\text{d}x(t,\alpha ,\epsilon )}{\text{d}t}=u(x(t,\alpha ,\epsilon ),t,\alpha ,\epsilon ),$$

where α is a wave phase, regarded as an ensemble parameter. The fluid velocity u(x,t,α,ϵ) is incompressible, ∇ ⋅ u=0, and has the form

2.2 $$u(x,t,\alpha ,\epsilon )=\epsilon u_{1}(x,t,\alpha )+\epsilon^{2}u_{2}(x,t,\alpha )+\cdots ,$$

where ϵ is the wave amplitude parameter. The leading-order term $u_{1}(x,t,\alpha )$ is a fast wavy flow, so $\bar{u_{1}}=0$, where the mean, denoted by the overbar, is an average over the phase α.

To average the fast wave oscillations in (2.1), we consider the *ansatz*

2.3*a* $$\begin{array}{rl} x(t,\alpha ,\epsilon ) & \;=x^{\text{L}}(t,\epsilon )+\xi (x^{\text{L}}(t,\epsilon ),t,\alpha ,\epsilon ), \end{array}$$

2.3*b* $$\begin{array}{rl} & \;=x^{\text{L}}(t,\epsilon )+\epsilon \xi_{1}(x^{\text{L}}(t,\epsilon ),t,\alpha )+\epsilon^{2}\xi_{2}(x^{\text{L}}(t,\epsilon ),t,\alpha )+\cdots , \end{array}$$

where $x^{\text{L}}(t,\epsilon )$ is the slow motion of a Lagrangian mean position. Think of $x^{\text{L}}$ as a ‘guiding centre’ such that rapid wavy oscillations are confined to the displacements $\xi_{n}$; these displacements from $x^{\text{L}}$ do not grow with time, i.e. all members of the ensemble remain close to the guiding centre $x^{\text{L}}$. The motion of $x^{\text{L}}$ is written as

2.4 $$\begin{array}{rl} \frac{\text{d}x^{\text{L}}}{\text{d}t} & \;=\epsilon^{2}u^{L}(x^{\text{L}},t) \end{array}$$

2.5 $$\begin{array}{rl} & \;=\epsilon^{2}u_{2}^{L}(x^{\text{L}},t)+\epsilon^{3}u_{3}^{L}(x^{\text{L}},t)+\cdots , \end{array}$$

where $u^{\text{L}}(x,t,\epsilon )$ is the Lagrangian mean velocity, yet to be defined and determined. The ansatz (2.3*b*) is ambiguous because requiring only that the $\xi_{n}$’s do not grow with time does not uniquely determine $u^{\text{L}}$ and $\xi_{n}$. We return to this point below.

Substituting (2.3*b*) and (2.5) into (2.1) and (2.2) and matching powers of ϵ at the first two orders results in

2.6*a* $$\partial_{t}\xi_{1}(x^{\text{L}},t,\alpha )=u_{1}(x^{\text{L}},t,\alpha )$$

and

2.6*b* $$u_{2}^{L}(x^{\text{L}},t)+\partial_{t}\xi_{2}(t,\alpha )=u_{2}(x^{\text{L}},t,\alpha )+\xi_{1}(x^{\text{L}},t,\alpha )\;\cdot \;\nabla u_{1}(x^{\text{L}},t,\alpha ).$$

Choosing to follow Stokes [1] and Andrews & McIntyre [8], one disambiguates (2.3*b*) by requiring that $\bar{\xi_{2}(x^{\text{L}},t,\alpha )}=0$. In this case, averaging (2.6*b*) produces the familiar result

2.7 $$u^{L}=\underset{u^{E}}{\underbrace{\bar{u_{2}}}}+\underset{u^{\text{S}}}{\underbrace{\bar{\left(\xi_{1}\;\cdot \;\nabla \right)\;u_{1}}}}\;(\text{with }\bar{\xi_{2}}=0).$$

In (2.7) we have now omitted the subscript 2 on $u^{\text{L}}$. With (2.7) we recover (1.2) and the small wave-amplitude version of (1.1).

### (b) The solenoidal Stokes velocity $u_{\mathrm{sol}}^{S}$

Let us examine the divergence of $u^{\text{S}}$ in more detail. The ‘un-averaged Stokes velocity’ can be written exactly as

2.8*a* $$\begin{array}{rl} \xi_{1}\;\cdot \;\nabla u_{1} & \;=\partial_{t}\frac{1}{2}\left(\xi_{1}\;\cdot \;\nabla \right)\xi_{1}+\frac{1}{2}\left(\xi_{1}\;\cdot \;\nabla \right)u_{1}-\frac{1}{2}\left(u_{1}\;\cdot \;\nabla \right)\xi_{1}, \end{array}$$

2.8*b* $$\begin{array}{rl} & \;=\partial_{t}\frac{1}{2}\left(\xi_{1}\;\cdot \;\nabla \right)\xi_{1}+\nabla \times \frac{1}{2}(u_{1}\times \xi_{1}). \end{array}$$

In passing from (2.8*a*) to (2.8*b*), we have used wave incompressibility

2.9 $$\nabla \;\cdot \;u_{1}=\nabla \;\cdot \;\xi_{1}=0,$$

to simplify the standard vector identity for the curl of the cross product $u_{1}\times \xi_{1}$. The average of (2.8*b*),

2.10 $$u^{\text{S}}=\underset{O(\epsilon^{2}\mu )}{\underbrace{\partial_{t}\frac{1}{2}\bar{\left(\xi_{1}\;\cdot \;\nabla \right)\xi_{1}}}}+\underset{O(\epsilon^{2})}{\underbrace{\nabla \times \frac{1}{2}(\bar{u_{1}\times \xi_{1}})}},$$

identifies a solenoidal part of the Stokes velocity as

2.11*a* $$\begin{array}{rl} u_{\text{sol}}^{\text{S}} & \;\overset{\text{def}}{=}\nabla \times \left(\frac{1}{2}\;\bar{u_{1}\times \xi_{1}}\right), \end{array}$$

2.11*b* $$\begin{array}{rl} & \;=\frac{1}{2}\bar{\left(\xi_{1}\;\cdot \;\nabla \right)\;u_{1}}-\frac{1}{2}\bar{\left(u_{1}\;\cdot \;\nabla \right)\;\xi_{1}}. \end{array}$$

The solenoidal vector $u_{\text{sol}}^{\text{S}}$ is the incompressible part of the Stokes velocity for all types of weakly nonlinear waves in an incompressible fluid. We propose that $u_{\text{sol}}^{\text{S}}$ can advantageously replace the traditional form of the Stokes velocity (1.2) in many circumstances.

The solenoidal Stokes velocity arises naturally if a small change is made in the derivation in §2(a): suppose we decide from the outset to change the definition of ‘Lagrangian mean’ so that $u^{\text{L}}$ in (2.5) is solenoidal. To implement this choice, we disambiguate (2.3*b*) with $\bar{\xi_{2}}=\frac{1}{2}\bar{\left(\xi_{1}\;\cdot \;\nabla \right)\xi_{1}}$ (rather than $\bar{\xi_{2}}=0$). Averaging (2.6*b*) then results in

2.12 $$u^{L}=\underset{u^{E}}{\underbrace{\bar{u_{2}}}}+\underset{u_{\text{sol}}^{S}}{\underbrace{\nabla \times \left(\frac{1}{2}\bar{u_{1}\times \xi_{1}}\right)}},\;(\text{with }\bar{\xi_{2}}=\frac{1}{2}\bar{\left(\xi_{1}\;\cdot \;\nabla \right)\xi_{1}}).$$

The incompressible Lagrangian mean velocity in (2.12) is an alternative to the traditional compressible $u^{\text{L}}$ in (2.7). The $O(\epsilon^{2})$ shift $\bar{\xi_{2}}$ in the position of guiding centre $x^{\text{L}}(t,\epsilon )$ produces Lagrangian mean and Stokes velocities that are divergence free at order $\epsilon^{2}$. In §4, we discuss a systematic framework—Soward & Robert’s [18] glm alternative to GLM—that generalizes this property to arbitrarily high order in ϵ.

Note that the difference between $u^{\text{S}}$ and $u_{\text{sol}}^{\text{S}}$ is a time derivative so has no impact on particle dispersion for waves that are represented by stationary random processes as discussed by Holmes-Cerfon & Bühler [22].

### (c) Irrotational linear waves

In addition to incompressibility in (2.9), surface gravity waves are also irrotational,

2.13 $$\nabla \times u_{1}=\nabla \times \xi_{1}=0.$$

Using (2.13), the final term in the vector identity $\xi_{1}\cdot \nabla \xi_{1}=\nabla \frac{1}{2}|\xi_{1}{|}^{2}+(\nabla \times \xi_{1})\times \xi_{1}$ is zero and (2.10) simplifies further to a Helmholtz decomposition of the surface-wave Stokes velocity

2.14 $$u^{\text{S}}=\partial_{t}\nabla \frac{1}{4}\bar{|\xi_{1}{|}^{2}}+\underset{u_{\text{sol}}^{\text{S}}}{\underbrace{\nabla \times \frac{1}{2}\bar{u_{1}\times \xi_{1}}}}.$$

The divergence of the surface-wave Stokes velocity in (2.14) is

2.15 $$\nabla \;\cdot \;u^{\text{S}}=\underset{O(\epsilon^{2}\mu )}{\underbrace{\partial_{t}△\frac{1}{4}\bar{|\xi_{1}{|}^{2}}}},$$

where $△=\partial_{x}^{2}+\partial_{y}^{2}+\partial_{z}^{2}$ is the Laplacian. The expression for $\nabla \;\cdot \;u^{\text{S}}$ in (2.15) is simpler than that in (1.3), but restricted to waves with $\nabla \times \xi_{1}=0$.

The curl of the Stokes velocities does not vanish:

2.16 $$\begin{array}{rl} \nabla \times u^{\text{S}}=\nabla \times u_{\text{sol}}^{\text{S}} & \;=\nabla \times \nabla \times \frac{1}{2}\bar{u_{1}\times \xi_{1}}, \end{array}$$

2.17 $$\begin{array}{rl} & \;=-△\frac{1}{2}\bar{u_{1}\times \xi_{1}}, \end{array}$$

where we have used ∇×∇×=∇∇ ⋅ −△ and $\nabla \;\cdot \;\frac{1}{2}\bar{u_{1}\times \xi_{1}}=0$. With an error of order $\mu^{2}\epsilon^{2}$, we can replace △ with $\partial_{z}^{2}$ in (2.17).

## 3. Stokes transport and Stokes pumping

Discussions of the deep return flow associated with a surface-gravity wave packet argue [2] that:

‘the return flow ⋯ can be explained as the irrotational response to balance the Stokes transport ⋯ that acts to “pump” fluid from the trailing edge of the packet to the leading edge’.

Similar sentiments are expressed in [23,24]. With this physical picture in mind, it is instructive to compare the Stokes pumping associated with $u^{\text{S}}$ with that of $u_{\text{sol}}^{\text{S}}$. The interpretation of the return flow in the quotation above is most consistent with $u_{\text{sol}}^{\text{S}}$.

### (a) Stokes pumping and $u_{\mathrm{sol}}^{S}=\nabla \times \frac{1}{2}\bar{u_{1}\times \xi_{1}}$

We start with the easy solenoidal case, with Stokes transport

3.1 $$T_{\text{sol}}^{\text{S}}(x,y,t)\overset{\text{def}}{=}\int_{-\infty }^{0}\;(u_{\text{sol}}^{\text{S}}\;\hat{x}+v_{\text{sol}}^{\text{S}}\;\hat{y})\;\text{d}z,$$

where the vertical integration above is from the bottom of the wave-active zone (denoted −∞) to the mean sea surface at z=0. (In this section, we confine attention to deep-water waves so that the lower limit, −∞, is well above the distant bottom.) Vertical integration of $\nabla \;\cdot \;u_{\text{sol}}^{\text{S}}=0$ over the wave-active region produces the unsurprising result

3.2*a* $$\begin{array}{rl} \text{solenoidal Stokes pumping} & \;\overset{\text{def}}{=}-\nabla \;\cdot \;T_{\text{sol}}^{\text{S}}, \end{array}$$

3.2*b* $$\begin{array}{rl} & \;=w_{\text{sol}}^{\text{S}}{|}_{0}, \end{array}$$

where we use ${|}_{0}$ to denote evaluation at z=0, e.g. $w_{\text{sol}}^{\text{S}}{|}_{0}=w_{\text{sol}}^{\text{S}}(x,y,0,t)$. The result (3.2*b*) is consistent with the idea that horizontal convergence within the wave-active zone pumps fluid downwards, out of the wave-active zone, with the vertical velocity $w_{\text{sol}}^{\text{S}}(x,y,0,t)$.

Vertical integration of $u_{\text{sol}}^{\text{S}}=\frac{1}{2}\bar{(\xi_{1}\cdot \nabla )u_{1}}-\frac{1}{2}\bar{(u_{1}\cdot \nabla )\xi_{1}}$ over the wave-active zone results in

3.3*a* $$\hat{x}\;\cdot \;T_{\text{sol}}^{\text{S}}=\frac{1}{2}(\bar{\zeta_{1}u_{1}}-\bar{w_{1}\xi_{1}}){|}_{0}+\partial_{y}\chi \;$$

and

3.3*b* $$\hat{y}\;\cdot \;T_{\text{sol}}^{\text{S}}=\frac{1}{2}(\bar{\zeta_{1}v_{1}}-\bar{w_{1}\eta_{1}}){|}_{0}-\partial_{x}\chi ,$$

where

3.4 $$\chi \;\overset{\text{def}}{=}\;\frac{1}{2}\int_{-\infty }^{0}(\bar{\eta_{1}u_{1}}-\bar{v_{1}\xi_{1}})\;\text{d}z$$

is a streamfunction for horizontally circulating Stokes transport. This horizontal Stokes circulation is previously unremarked, perhaps because the terms involving χ in (3.3*a*) and (3.3*b*) are order μ smaller than the other terms. Using the coordinate expressions in (3.3*a*) and (3.3*b*), the solenoidal pumping can be expressed entirely in terms of surface quantities:

3.5 $$\text{solenoidal Stokes pumping}=\partial_{x}\frac{1}{2}(\bar{\zeta_{1}u_{1}}-\bar{w_{1}\xi_{1}}){|}_{0}+\partial_{y}\frac{1}{2}(\bar{\zeta_{1}v_{1}}-\bar{w_{1}\eta_{1}}){|}_{0}.$$

### (b) Stokes pumping and $u^{S}=\bar{(\xi_{1}\;\cdot \;\nabla )u_{1}}$

We turn now to the traditional definition of the Stokes velocity. Define the Stokes transport via

3.6 $$T^{\text{S}}(x,y,t)\overset{\text{def}}{=}\int_{-\infty }^{0}(u^{\text{S}}\;\hat{x}+v^{\text{S}}\;\hat{y})\;\text{d}z,$$

and the Stokes pumping by

3.7 $$\text{Stokes pumping}\overset{\text{def}}{=}-\nabla \;\cdot \;T^{\text{S}}.$$

Because $\nabla \;\cdot \;u^{\text{S}}\neq 0$ there is no analogy to (3.2*b*). Instead, integrating (2.15) over the wave-active zone

3.8 $$\nabla \;\cdot \;T^{\text{S}}+w^{\text{S}}{|}_{0}=\partial_{t}\partial_{z}\frac{1}{4}\bar{|\xi_{1}{|}^{2}}{|}_{0}+\partial_{t}(\partial_{x}^{2}+\partial_{y}^{2})\int_{-\infty }^{0}\frac{1}{4}|\xi_{1}{|}^{2}\;\text{d}z.$$

With vertical integration of the horizontal components of $u^{\text{S}}$ over the wave-active zone

3.9*a* $$\hat{x}\cdot T^{\text{S}}=(\bar{\zeta_{1}u_{1}}){|}_{0}+\partial_{t}\partial_{x}\int_{-\infty }^{0}\frac{1}{2}\bar{\xi_{1}^{2}}\;\text{d}z+\partial_{y}\int_{-\infty }^{0}\bar{\eta_{1}u_{1}}\;\text{d}z$$

and

3.9*b* $$\hat{y}\;\cdot \;T^{\text{S}}=(\bar{\zeta_{1}v_{1}}){|}_{0}+\partial_{x}\int_{-\infty }^{0}\bar{\xi_{1}v_{1}}\;\text{d}z+\partial_{t}\partial_{y}\int_{-\infty }^{0}\frac{1}{2}\bar{\eta_{1}^{2}}\text{d}z.$$

The horizontal divergence of $T^{\text{S}}$ is therefore

3.10 $$\nabla \;\cdot \;T^{\text{S}}=\partial_{x}(\bar{\zeta_{1}u_{1}}){|}_{0}+\partial_{y}(\bar{\zeta_{1}v_{1}}){|}_{0}+\partial_{t}(\frac{1}{2}\int_{-\infty }^{0}\xi_{1,\alpha }\xi_{1,\beta }\;\text{d}z{)}_{,\alpha \beta }.$$

In the final term, the indices α and β are 1 and 2. Eliminating $\nabla \;\cdot \;T^{\text{S}}$ between (3.8) and (3.10)

3.11 $$\begin{array}{rl} w^{\text{S}}{|}_{0} & \;=-\partial_{x}(\bar{\zeta_{1}u_{1}}){|}_{0}-\partial_{y}(\bar{\zeta_{1}v_{1}}){|}_{0}+\partial_{t}\partial_{z}\frac{1}{4}\bar{|\xi_{1}{|}^{2}}{|}_{0}\\ & \;\;+\partial_{t}(\partial_{x}^{2}+\partial_{y}^{2})\int_{-\infty }^{0}\frac{1}{4}|\xi_{1}{|}^{2}\;\text{d}z-\partial_{t}(\frac{1}{2}\int_{-\infty }^{0}\xi_{1,\alpha }\xi_{1,\beta }\;\text{d}z{)}_{,\alpha \beta }. \end{array}$$

The results in (3.8), (3.10) and (3.11) are all more complicated than their solenoidal cousins in (3.2*b*), (3.3*a*) and (3.3*b*).

### (c) A comment on approximations to the Stokes transport

A widely used expression for the Stokes transport is

3.12 $$T^{\text{S}}\approx \bar{\left(\zeta_{1}u_{1}\right)}{|}_{0}.$$

Expression (3.12) is exact for a uniform (μ=0) progressive wave and is a leading-order approximation for a slowly modulated (μ≪1) wave packet. To see the connection with the more general and exact results above, align the x-axis with the horizontal wavevector so that $\eta_{1}=v_{1}=0$; then $\hat{y}\;\cdot \;T^{\text{S}}=\hat{y}\;\cdot \;T_{\text{sol}}^{\text{S}}=\chi =0$. Thus, with an error by order $\epsilon^{2}\mu$, and in agreement with (3.12),

3.13 $$\frac{1}{2}(\bar{\zeta_{1}u_{1}}-\bar{w_{1}\xi_{1}}){|}_{0}\approx \bar{\left(\zeta_{1}u_{1}\right)}{|}_{0}.$$

In other words, (3.12) is a leading-order approximation and at this order $T^{\text{S}}$ and $T_{\text{sol}}^{\text{S}}$ are identical.

## 4. glm

We return to the definition of the Stokes velocity and show how the glm theory of Soward & Roberts [18] rationalizes and generalizes the heuristic construction of the solenoidal velocity $u_{\text{sol}}^{\text{S}}$. In §2(a), we emphasized the ambiguity in the decomposition (2.3a) of trajectories into a mean part $x^{\text{L}}$ and a perturbation ξ. GLM resolves this ambiguity by imposing that

4.1 $$\bar{\xi }=0.$$

While (4.1) is widely accepted, it is neither inevitable nor particularly natural. It implies that the Lagrangian-mean trajectory of a particle is defined by the equality $x^{\text{L}}=\bar{x}$ between the coordinates of the Lagrangian-mean position and the average of the coordinates of the particle. This indicates that the Lagrangian-mean trajectory and, as a result, the Lagrangian-mean velocity $u^{\text{L}}$ depend on a choice of coordinates. This undesirable feature of GLM is remedied by glm, while also ensuring that $u^{\text{L}}$ is non-divergent (see [21] for other alternatives to GLM).

### (a) Formulation

To introduce glm, it is convenient to rewrite equation (2.1) governing the fluid trajectories in terms of the flow map φ(x,t,α,ϵ) giving the position at time t of the fluid particle initially at x. Equation (2.1) becomes

4.2 $$\dot{\varphi }(x,t,\alpha ,\epsilon )=u(\varphi (x,t,\alpha ,\epsilon ),t,\alpha ,\epsilon ).$$

The decomposition of trajectories into mean and perturbation is best written as the composition

4.3 $$\varphi =\Xi \circ \varphi^{\text{L}},$$

or, more explicitly, $\varphi (x,t,\alpha ,\epsilon )=\Xi (\varphi^{\text{L}}(x,t,\epsilon ),t,\alpha ,\epsilon )$. Here $\varphi^{\text{L}}$ is the (α-independent) mean map, sending the initial position of particles to their mean position, and Ξ is the perturbation map, sending the mean position to the exact, perturbed position. The perturbation map can only be represented in the familiar form x↦x+ξ(x,t,α,ϵ), as in (2.3a), in Euclidean space, where positions x can be identified with vectors and added, or, in more general geometries, once a specific coordinate system has been chosen and x is interpreted as a triple of coordinates. The smallness of the perturbation, usually stated as |ξ|≪1, translates into the requirement that Ξ is close to the identity map. We emphasize that the mean map $\varphi^{\text{L}}$ is not obtained from φ by applying an averaging operator: averaging is a linear operation that applies to linear objects, such as vector fields, but not to nonlinear maps such as φ. Instead, $\varphi^{\text{L}}$ is defined by imposing a condition on the perturbation map Ξ. The form of the Lagrangian-mean velocity $u^{\text{L}}$, defined by

4.4 $$\dot{\varphi^{\text{L}}}(x,t,\epsilon )=\epsilon^{2}u^{L}(\varphi^{\text{L}}(x,t),t,\epsilon ),\;\text{i.e.}\;\epsilon^{2}u^{L}=\dot{\varphi^{\text{L}}}\circ {\left(\varphi^{\text{L}}\right)}^{-1},$$

depends on this condition.

The defining condition of glm is expressed as follows. The small parameter ϵ is regarded as a fictitious time, and the perturbation map Ξ is constructed as the flow at ‘time’ ϵ of a vector field, q say; that is, Ξ(x,t,α,ϵ) is the solution of

4.5 $$\partial_{\epsilon }\Xi (x,t,\alpha ,\epsilon )=q(\Xi (x,t,\alpha ,\epsilon ),t,\alpha ,\epsilon )\;\text{with}\;\Xi (x,t,\alpha ,\epsilon =0)=x,$$

and t is treated as a fixed parameter. The glm condition is then

4.6 $$\bar{q}=0.$$

Although superficially similar to the GLM condition (4.1), (4.6) is fundamentally different in that it is an intrinsic statement, applicable to any manifold and independent of any coordinate choice. Moreover, the glm formulation defines an exactly divergence-free Lagrangian mean flow, by requiring that

4.7 ∇ ⋅ q=0

to ensure that Ξ and $\varphi^{\text{L}}$ preserve volume and hence $\nabla \;\cdot \;u^{\text{L}}=0$.

Equation (4.6) generalizes the condition on $\bar{\xi_{2}}$ in (2.12) which leads to $u_{\text{sol}}^{\text{S}}$ as the Stokes velocity. We show this by solving (4.5) by Taylor expansion. In coordinates, we have that

4.8 $$\Xi (x,t,\alpha ,\epsilon )=x+\xi (x,t,\alpha ,\epsilon )=x+\epsilon q(x,t,\alpha ,0)+\frac{1}{2}\epsilon^{2}(\partial_{\epsilon }q+q\;\cdot \;\nabla q)(x,t,\alpha ,0)+\cdots .$$

The power series expansions (2.3b) for ξ and

4.9 $$q=q_{1}+\epsilon q_{2}+\epsilon^{2}q_{3}+\cdots$$

for q then give

4.10 $$\xi_{1}=q_{1}\;\text{and}\;\xi_{2}=q_{2}+\frac{1}{2}q_{1}\cdot \nabla q_{1}$$

so that (4.6) implies (2.12). In the next section, we develop a systematic computation of $u^{\text{L}}$ and hence $u_{\text{sol}}^{\text{S}}$ order by order in ϵ using Lie series. This removes the need to introduce ξ by focusing the perturbation expansion on q.

### (b) Lie series expansion

The glm formalism can be regarded as an instance of classical perturbation theory, which approximates solutions to the ordinary differential equation (4.2) by performing a variable transformation designed to eliminate fast time dependences. In this interpretation, Ξ determines the variable transformation and $\varphi^{\text{L}}$ represents the new variable. Lie series [19,20] provide a powerful tool for the systematic implementation of classical perturbation theory which we now apply to glm.

Introducing the decomposition (4.3) into (4.2) and using (4.4) gives

4.11 $$w+\Xi {\;}_{*}\;u^{L}=u,$$

where

4.12 $$w=\dot{\Xi }\circ \Xi^{-1}$$

is the perturbation velocity and $\Xi_{*}$ is the push-forward by Ξ, with $\Xi_{\;*\;}u^{\text{L}}=(u^{\text{L}}\;\cdot \;\nabla )\Xi$ in Cartesian coordinates. Pulling back (4.11) gives

4.13 $$u^{L}=\Xi^{*}u-\hat{w},\;\text{where}\;\hat{w}=\Xi^{*}w.$$

We seek an ϵ-dependent Ξ to eliminate fast time dependence from $u^{\text{L}}$ order-by-order in ϵ, and formulate the problem in terms of the vector field q that generates Ξ according to (4.5). We impose that ∇⋅q=0 to ensure that $\nabla \;\cdot \;u^{\text{L}}=0$, and the glm condition (4.6).

Expanding q as in (4.9) we relate the various terms by differentiating (4.13) repeatedly with respect to ϵ and evaluating the results at ϵ=0. Two key identities turn this into a mechanical exercise. The first, essentially the definition of the Lie derivative [25], is

4.14 $$\partial_{\epsilon }\Xi^{*}u=\Xi^{*}L_{q}u,$$

where $L_{q}u=q\;\cdot \;\nabla u-u\;\cdot \;\nabla q$ is the Lie derivative of u along q. The second,

4.15 $$\partial_{\epsilon }\hat{w}=\partial_{t}(\Xi^{*}q)+L_{\Xi^{*}q}\hat{w},$$

is established in appendix A. Iterating (4.14) and (4.15) we find

4.16 $$\begin{array}{rl} \Xi^{*}u & \;=u+\epsilon L_{q_{1}}u+\frac{1}{2}\epsilon^{2}(L_{q_{1}}^{2}+L_{q_{2}})u+\frac{1}{6}\epsilon^{3}(L_{q_{1}}^{3}+2L_{q_{1}}L_{q_{2}}+L_{q_{2}}L_{q_{1}}+2L_{q_{3}})u+\cdots \end{array}$$

and

4.17 $$\begin{array}{rl} \hat{w} & \;=\epsilon \partial_{t}q_{1}+\frac{1}{2}\epsilon^{2}(\partial_{t}q_{2}+L_{q_{1}}\partial_{t}q_{1})+\frac{1}{3}\epsilon^{3}(\partial_{t}q_{3}+L_{q_{1}}\partial_{t}q_{2}+\frac{1}{2}L_{q_{2}}\partial_{t}q_{1}+\frac{1}{2}L_{q_{1}}^{2}\partial_{t}q_{1})+\cdots \end{array}$$

Introducing (4.16) and (4.17) into (4.13) gives at the first two orders in ϵ,

4.18 $$\partial_{t}q_{1}=u_{1},\;\text{i.e.}\;q_{1}=\xi_{1}$$

and

4.19 $$u^{L}={\bar{u}}_{2}+\frac{1}{2}\bar{L_{q_{1}}u_{1}},$$

on using (4.6). This provides the geometric formula

4.20 $$u_{\text{sol}}^{\text{S}}=\frac{1}{2}\bar{L_{q_{1}}u_{1}}$$

for the solenoidal Stokes drift, equivalent to (2.11*a*) since $L_{q_{1}}u_{1}=q_{1}\cdot \nabla u_{1}-u_{1}\cdot \nabla q_{1}=\nabla \times (u_{1}\times \xi_{1})$. The expansion can be pursued to higher orders by choosing divergence-free $q_{n}$ that push the fast dependence on the right-hand side of (4.13) to order $\epsilon^{n+1}$. This yields $u^{\text{L}}$ and hence $u_{\text{sol}}^{\text{S}}$ to arbitrary order in ϵ.

### (c) Lagrangian-mean momentum equation

A Lagrangian-mean momentum equation, analogous to Andrews & McIntyre Theorem I [8], can be derived for the glm formalism order-by-order in ϵ [18,21]. We sketch a derivation, focusing on the case of small-amplitude surface gravity waves. This derivation is conveniently carried out using a representation of the rotating Euler equation

4.21 $$D_{t}u+f\times u=-\nabla p,$$

with $D_{t}=\partial_{t}+u\;\cdot \;\nabla$, in terms of the absolute momentum 1-form [21,26,27]

4.22 $$\nu_{a}=u\;\cdot \;\text{d}x+\frac{1}{2}(f\times x)\;\cdot \;\text{d}x.$$

It can be checked that (4.21) is equivalent to

4.23 $$(\partial_{t}+L_{u})\nu_{a}=-\text{d}\pi ,$$

where $\pi =p-\frac{1}{2}|u{|}^{2}-\frac{1}{2}(f\times x)\cdot u$, using basic properties of the Lie derivative, namely Leibniz rule, commutation with the differential d, and that $L_{u}=u\cdot \nabla$ when applied to scalars [25]; see appendix A for details. Equation (4.23) can be thought of as a local version of Kelvin’s circulation theorem. This is readily obtained by integration along a closed curve C(t) moving with u to find

4.24 $$\oint_{C(t)}\nu_{a}=\oint_{C(t)}(u+\frac{1}{2}(f\times x))\;\cdot \;\text{d}x=\text{const}.$$

An advantage of (4.23) is that it leads directly to a Lagrangian-mean momentum equation of a similar form [21],

4.25 $$(\partial_{t}+L_{u^{L}})\nu_{a}^{\text{L}}=-\text{d}\pi^{\text{L}},$$

and to the corresponding Lagrangian-mean Kelvin’s circulation theorem

4.26 $$\oint_{C^{\text{L}}(t)}\nu_{a}^{\text{L}}=\text{const},$$

where the closed curve $C^{\text{L}}(t)$ moves with the Lagrangian-mean velocity $u^{\text{L}}$. Here the Lagrangian-mean momentum and effective pressure are given by

4.27 $$\nu_{a}^{\text{L}}=\bar{\Xi^{*}\nu_{a}}\;\text{and}\;\pi^{\text{L}}=\bar{\Xi^{*}\pi }.$$

The pull-back $\Xi^{*}$ by the perturbation map acts on scalars as a composition, e.g. $\left(\Xi^{*}\pi )(x,t,\alpha ,\epsilon )=\pi (\Xi (x,t,\alpha ,\epsilon ),t,\alpha ,\epsilon \right)$, and commutes with the differential so that

4.28 $$(\Xi^{*}(u\;\cdot \;\text{d}x))(x)=u(\Xi (x))\;\cdot \;\text{d}\Xi (x)=u(\Xi (x))\cdot (\text{d}x\;\cdot \;\nabla )\Xi (x).$$

We show in appendix A that (4.25), together with the coordinate representation Ξ(x,t,α,ϵ)=x+ξ(x,t,α,ϵ) and GLM condition (4.1) recovers Andrews & McIntyre’s Lagrangian-mean momentum equation [8, theorem I]. For glm, we can use the Lie-series expression (4.16) to obtain an expansion of $\nu_{a}^{\text{L}}$ as

4.29 $$\nu_{a}^{\text{L}}=\frac{1}{2}(f\times x)\;\cdot \;\text{d}x+\epsilon^{2}\;\bar{u_{2}\cdot \text{d}x+L_{q_{1}}(u_{1}\cdot \text{d}x)+\frac{1}{4}L_{q_{1}}^{2}((f\times x)\;\cdot \;\text{d}x)}+O(\epsilon^{3})$$

using that $u=\epsilon u_{1}+\epsilon^{2}u_{2}+\cdots$ and $\bar{q_{1}}=\bar{q_{2}}=0$. Introducing (4.29) into (4.25) and expanding the Lie derivative gives an evolution equation for the Eulerian mean velocity $u^{\text{E}}=\bar{u_{2}}$ supplemented by the incompressibility condition $\nabla \;\cdot \;u^{\text{E}}=0$.

The Lagrangian-mean momentum equation greatly simplifies for surface gravity waves: typical wave frequencies σ satisfy σ/f≫1 and we can therefore neglect the term involving $L_{q_{1}}^{2}$ in (4.29) against the other two in the average (recall that $\partial_{t}q_{1}=u_{1}$). Moreover, using the irrotationality condition (2.13), we compute

4.30 $$\begin{array}{rl} \bar{L_{q_{1}}(u_{1}\cdot \text{d}x)} & \;=\bar{(q_{1}\cdot \nabla )u_{1}\cdot \text{d}x+u_{1}\cdot \text{d}q_{1}}=\bar{\left(q_{1j}u_{1i,j}+u_{1j}q_{1j,i}\right)}\;\text{d}x_{i}\\ & \;=\bar{\left(q_{1j}u_{1j,i}+u_{1j}q_{1j,i}\right)}\text{d}x_{i}=\partial_{t}\bar{\left(q_{1j}q_{1j,i}\right)}\;\text{d}x_{i}\\ & \;=\frac{1}{2}\partial_{t}\nabla \bar{|q_{1}{|}^{2}}\cdot \text{d}x=\frac{1}{2}\text{d}(\partial_{t}\bar{|q_{1}{|}^{2}})=O(\mu \epsilon^{2}). \end{array}$$

Therefore, to leading order, the glm mean momentum equation reduces to

4.31 $$(\partial_{t}+L_{u^{L}})(u^{E}\;\cdot \;\text{d}x+\frac{1}{2}(f\times x)\;\cdot \;\text{d}x)=-\text{d}\pi^{\text{L}},$$

with the glm Lagrangian-mean velocity in (2.12) (see [26,27] for an analogous formulation of the GLM CL equation). Using Cartan’s formula in the form

4.32 $$L_{u}(v\;\cdot \;\text{d}x)=((\nabla \times v)\times u)\;\cdot \;\text{d}x+\text{d}(u\cdot v),$$

the Lie derivatives in (4.31) can be written as

4.33 $$\begin{array}{rl} L_{u^{L}}((f\times x)\cdot \text{d}x) & \;=2(f\times u^{L})\cdot \text{d}x+\text{d}(\cdot ), \end{array}$$

4.34 $$\begin{array}{rl} L_{u^{L}}(u^{E}\;\cdot \;\text{d}x) & \;=L_{u^{\text{E}}}(u^{E}\;\cdot \;\text{d}x)+L_{u_{\text{sol}}^{\text{S}}}(u^{E}\;\cdot \;\text{d}x)\\ & \;=(u^{\text{E}}\;\cdot \;\nabla u^{\text{E}})\;\cdot \;\text{d}x+((\nabla \times u^{\text{E}})\times u_{\text{sol}}^{\text{S}})\;\cdot \;\text{d}x+\text{d}(\cdot ), \end{array}$$

where we do not detail the exact differentials. This makes it possible to rewrite (4.31) as

4.35 $$\partial_{t}u^{E}+(u^{E}\;\cdot \;\nabla )u^{E}+\times (u^{E}+u_{\text{sol}}^{\text{S}})=-\nabla \varpi +u_{\text{sol}}^{\text{S}}\times (\nabla \times u^{E}),$$

for a suitable definition of the effective pressure ϖ. Equation (4.35) can be recognized as the CL equation [3,4,28] with the solenoidal Stokes velocity $u_{\text{sol}}^{\text{S}}$ replacing $u^{\text{S}}$. This is not surprising since the $O(\epsilon^{2}\mu )$ difference between $u_{\text{sol}}^{\text{S}}$ and $u^{\text{S}}$ is of the same order as terms neglected in the derivation of the CL equation. (CL geared μ to ϵ by taking $\mu =\epsilon^{2}$. In our derivation, this gearing is not necessary.) In fact the assumption μ≪1 is only used to neglect the term (4.30) from the Lagrangian-mean momentum equation to obtain (4.31) and hence (4.35). Restoring this term leads to the generalization

4.36 $$(\partial_{t}+L_{u^{L}})(u^{E}\;\cdot \;\text{d}x+\frac{1}{2}\text{d}(\partial_{t}\bar{|q_{1}{|}^{2}})+\frac{1}{2}(f\times x)\;\cdot \;\text{d}x)=-\text{d}\pi^{\text{L}},$$

of the CL equation valid for μ=O(1). Since $L_{u^{\text{L}}}$ and d commute, $L_{u^{\text{L}}}\text{d}(\partial_{t}\bar{|q_{1}{|}^{2}})=\text{d}(\cdots )$ and the additional terms involving $|q_{1}{|}^{2}$ can be absorbed in the differential of the pressure-like term on the right-hand side. We conclude that the CL equation (4.35) with $u_{\text{sol}}^{\text{S}}$ as Stokes velocity holds for μ=O(1) provided that the effective pressure ϖ is suitably redefined.

## 5. Conclusion

This paper examines a problematic aspect of the Stokes velocity $u^{\text{S}}$, namely its divergence $\nabla \;\cdot \;u^{\text{S}}$ and non-zero vertical component $w^{\text{S}}$. Some confusion has arisen because $\nabla \;\cdot \;u^{\text{S}}$ and $w^{\text{S}}$ are small when the wave field has an amplitude that varies on scales longer and slower than the wavelength and period. This scale-separation approximation corresponds to the existence of a small parameter μ≪1, as is the case for slowly varying wavepackets. The distinction between approximate and exact results in the literature is not always made plain. Beyond this, there are no irretrievable difficulties with the familiar form (1.2) of $u^{\text{S}}$: when $\nabla \;\cdot \;u^{\text{S}}$ and $w^{\text{S}}$ cannot be neglected, they are readily computed in terms of the first-order fields.

The main point of the paper, however, is that the Stokes velocity, understood as the difference $u^{\text{S}}=u^{\text{L}}-u^{\text{E}}$ between Lagrangian- and Eulerian-mean velocities, is not uniquely defined and that an alternative version, $u_{\text{sol}}^{\text{S}}$ in (2.11), that is exactly solenoidal can serve as a convenient substitute for the familiar (1.2). The non-uniqueness arises because there is no single definition of the Lagrangian-mean velocity $u^{\text{L}}$, which is only constrained to serve as a good ‘guiding-centre’ representative of the motion of an ensemble of fluid particles. The ambiguity is usually resolved by imposing the GLM condition $\bar{\xi }=0$, which is restricted to Euclidean geometry or depends on coordinates, but alternatives which have the benefit of both coordinate-independence and non-divergent $u^{\text{L}}$ exist [21]. One of these, Soward & Roberts’ glm [18], leads to $u_{\text{sol}}^{\text{S}}$ in (2.11) or, in a more geometric form, (4.20). We give only the form of $u_{\text{sol}}^{\text{S}}$ to leading order in the wave amplitude parameter ϵ; as we describe, higher-order corrections can be computed systematically using a Lie series expansion. We further show that the CL equations [3,4], widely used to model the feedback of surface gravity waves on the flow, apply in the glm framework, with $u_{\text{sol}}^{\text{S}}$ simply replacing $u^{\text{S}}$.

We stress that, in general, the coordinates associated with a Lagrangian-mean trajectory $x^{\text{L}}=\varphi^{\text{L}}(x_{0},t,\epsilon )$ of given fluid particle (identified by initial position $x_{0}$) are not the average of the coordinates of this particle. This is a feature specific to GLM, intimately connected to its coordinate dependence and the decision to make $\bar{\xi }=0$.

We conclude by noting that the assumption μ≪1, which makes it possible to ignore the divergence and vertical component of $u^{\text{S}}$, is restrictive. The assumption clearly holds for single wavepackets, but may fail for more complex wave fields. When it does, using the solenoidal Stokes velocity $u_{\text{sol}}^{\text{S}}$ brings considerable simplifications, e.g. for the computation of the Eulerian mean flow from the CL or other wave-averaged equations.

## Data Availability

This article has no additional data.

## Appendix A. Computational details for §4

**(a) Derivation of (4.15)**

We define $\hat{q}=\Xi^{*}q$ in analogy with $\hat{w}=\Xi^{*}w$ and note that both are the negative of velocities associated with the inverse map $\Xi^{-1}$ in the sense that

A 1 $$\partial_{t}\Xi^{-1}(x,t,\epsilon )=-\hat{w}(\Xi (x,t,\epsilon ),t,\epsilon )\;\text{and}\;\partial_{\epsilon }\Xi^{-1}(x,t,\epsilon )=-\hat{q}(\Xi (x,t,\epsilon ),t,\epsilon ),$$

as follows from the differentiation of the identity $\Xi^{-1}(\Xi (x,t,\epsilon ),t,\epsilon )=x$ with respect to t and ϵ. (We do not make the dependence on α explicit for simplicity.) Equating the derivative of the first equality with respect to ϵ with the derivative of the second with respect to t gives

A 2 $$-\partial_{\epsilon }\hat{w}+\hat{q}\;\cdot \;\nabla \hat{w}=-\partial_{t}\hat{q}+\hat{w}\;\cdot \;\nabla \hat{q}$$

and, on rearranging,

A 3 $$\partial_{\epsilon }\hat{w}=\partial_{t}\hat{q}+\hat{q}\;\cdot \;\nabla \hat{w}-\hat{w}\;\cdot \;\nabla q=\partial_{t}\hat{q}+L_{\hat{q}}\hat{w},$$

i.e. (4.15).

**(b) Equivalence between (4.21) and (4.23)**

We show that (4.23) is equivalent to the familiar form (4.21) of the Euler equation. We first write (4.23) explicitly as

A 4 $$(\partial_{t}+L_{u})(u\;\cdot \;\text{d}x+\frac{1}{2}(f\times x)\;\cdot \;\text{d}x)=-\text{d}(p-\frac{1}{2}|u{|}^{2}-\frac{1}{2}(f\times x)\;\cdot \;u).$$

Expanding, we find

A 5 $$\begin{array}{rl} & \;(D_{t}u)\;\cdot \;\text{d}x+u\;\cdot \;\text{d}u+\frac{1}{2}(f\times u)\;\cdot \;\text{d}x+\frac{1}{2}(f\times x)\;\cdot \;\text{d}u\\ & \;\;=-\nabla p\cdot \text{d}x+u\;\cdot \;\text{d}u+\frac{1}{2}(f\times \text{d}x)\;\cdot \;u+\frac{1}{2}(f\times x)\;\cdot \;\text{d}u, \end{array}$$

which recovers (4.21) since (f×dx)⋅u=−(f×u)⋅dx.

**(c) GLM from (4.25)**

We show that (4.25), together with the defining property of GLM $\bar{\xi }=0$ is equivalent to Theorem I of Andrews & McIntyre [8] in the case of an incompressible fluid. We need to apply the pull-back $\Xi^{*}$ to

A 6 $$\nu_{a}=u\;\cdot \;\text{d}x+\frac{1}{2}(f\times x)\;\cdot \;\text{d}x\;\text{and}\;\pi =p-\frac{1}{2}|u{|}^{2}-\frac{1}{2}(f\times x)\;\cdot \;u,$$

then average. Here u stands for the three components of u rather than for a vector, hence the pull-back is simply composition with Ξ, that is, $\Xi^{*}u=u\circ \Xi =:u^{\xi }$. We also have $\Xi^{*}x=x+\xi (x,t,\epsilon )$ and $\Xi^{*}\text{d}x=\text{d}(\Xi^{*}x)=\text{d}x+\text{d}\xi$. Using that $\bar{\xi }=0$, we compute

A 7 $$\nu_{a}^{\text{L}}=\bar{u^{\xi }\;\cdot \;\text{d}x+u^{\xi }\;\cdot \;\text{d}\xi +\frac{1}{2}(f\times \xi )\;\cdot \;\text{d}\xi }+\frac{1}{2}(f\times x)\;\cdot \;\text{d}x.$$

The averaged term can be written as $(u^{\text{L}}-\text{p})\cdot \text{d}x$ with $u^{\text{L}}=\bar{u^{\xi }}$ and

A 8 $${\text{p}}_{i}=-\bar{\xi_{j,i}(u_{j}^{\xi }+\frac{1}{2}{\left(f\times \xi \right)}_{j})}$$

the pseudomomentum. Similarly,

A 9 $$\pi^{\text{L}}=\bar{p^{\xi }-\frac{1}{2}|u^{\xi }{|}^{2}-\frac{1}{2}(f\times \xi )\;\cdot \;u^{\xi }}-\frac{1}{2}(f\times x)\;\cdot \;u^{L}.$$

Introducing (A 7) and (A 9) into (4.25), we compute

A 10 $$\begin{array}{rl} & \;(\partial_{t}+L_{u^{L}})((u^{L}-\text{p})\;\cdot \;\;\text{d}x)+((\nabla \times \frac{1}{2}(f\times x))\times u^{L})\;\cdot \;\text{d}x+\text{d}(\frac{1}{2}(f\times x)\;\cdot \;u^{L})\\ & \;\;=-\nabla {\tilde{\pi }}^{\text{L}}\;\cdot \;\text{d}x+\text{d}(\frac{1}{2}(f\times x)\;\cdot \;u^{L}), \end{array}$$

where ${\tilde{\pi }}^{\text{L}}=\bar{p^{\xi }-\frac{1}{2}|u^{\xi }{|}^{2}-\frac{1}{2}(f\times \xi )\cdot u^{\xi }}$ and we have used Cartan’s formula given below (4.31). Since ∇×(f×x)=2f we finally obtain

A 11 $$D_{t}(u_{i}^{\text{L}}-{\text{p}}_{i})+(u_{j}^{\text{L}}-{\text{p}}_{j})u_{j,i}^{\text{L}}+{\left(f\times u^{\text{L}}\right)}_{i}=-{\tilde{\pi }}_{,i}^{\text{L}},$$

that is, theorem I of [8].

## References

[1] Stokes GG. 1847 On the theory of oscillatory waves. Trans. Camb. Philos. Soc. **8**, 441-455.

[2] van den Bremer TS, Breivik Ø. 2018 Stokes drift. Phil. Trans. R. Soc. A **376**, 20170104. (10.1098/rsta.2017.0104) 29229803 PMC5740299

[3] Craik AD, Leibovich S. 1976 A rational model for Langmuir circulations. J. Fluid Mech. **73**, 401-426. (10.1017/S0022112076001420)

[4] Leibovich S. 1980 On wave-current interaction theories of Langmuir circulations. J. Fluid Mech. **99**, 715-724. (10.1017/S0022112080000857)

[5] Hasselmann K. 1970 Wave-driven inertial oscillations. Geophys. Astrophys. Fluid Dynam. **1**, 463-502. (10.1080/03091927009365783)

[6] Huang NE. 1979 On surface drift currents in the ocean. J. Fluid Mech. **91**, 191-208. (10.1017/S0022112079000112)

[7] McWilliams JC, Restrepo JM. 1999 The wave-driven ocean circulation. J. Phys. Oceanogr. **29**, 2523-2540. (10.1175/1520-0485(1999)029<2523:TWDOC>2.0.CO;2)

[8] Andrews DG, McIntyre ME. 1978 An exact theory of nonlinear waves on a Lagrangian-mean flow. J. Fluid Mech. **89**, 609-646. (10.1017/S0022112078002773)

[9] Bühler O. 2014 Waves and mean flows, 2nd edn. Cambridge, UK: Cambridge University Press.

[10] McIntyre ME. 1988 A note on the divergence effect and the Lagrangian-mean surface elevation in periodic water waves. J. Fluid Mech. **189**, 235-242. (10.1017/S0022112088000989)

[11] Phillips OM. 1977 The dynamics of the upper ocean. Cambridge, UK: Cambridge University Press.

[12] McWilliams JC, Restrepo JM, Lane EM. 2004 An asymptotic theory for the interaction of waves and currents in coastal waters. J. Fluid Mech. **511**, 135-178. (10.1017/S0022112004009358)

[13] Mellor G. 2016 On theories dealing with the interaction of surface waves and ocean circulation. J. Geophys. Res. Oceans **121**, 4474-4486. (10.1002/2016JC011768)

[14] van den Bremer TS, Whittaker C, Calvert R, Raby A, Taylor PH. 2019 Experimental study of particle trajectories below deep-water surface gravity wave groups. J. Fluid Mech. **879**, 168-186. (10.1017/jfm.2019.584)

[15] Monismith SG. 2019 Stokes drift: theory and experiments. J. Fluid Mech. **884**, F1. (10.1017/jfm.2019.891)

[16] Smith JA. 2006 Observed variability of ocean wave Stokes drift, and the Eulerian response to passing groups. J. Phys. Oceanogr. **36**, 1381-1402. (10.1175/JPO2910.1)

[17] Wagner GL, Chini GP, Ramadhan A, Gallet B, Ferrari R. 2021 Near-inertial waves and turbulence driven by the growth of swell. J. Phys. Oceanogr. **51**, 1337-1351. (10.1175/JPO-D-20-0178.1)

[18] Soward AM, Roberts PH. 2010 The hybrid Euler-Lagrange procedure using an extension of Moffatt’s method. J. Fluid Mech. **661**, 45-72. (10.1017/S0022112010002867)

[19] Lichtenberg A, Lieberman M. 1992 Regular and stochastic motion, 2nd edn. Berlin, Germany: Springer.

[20] Nayfeh AH. 2000 Perturbation methods. Weinheim, Germany: Wiley VCH.

[21] Gilbert AD, Vanneste J. 2018 Geometric generalised Lagrangian-mean theories. J. Fluid Mech. **839**, 95-134. (10.1017/jfm.2017.913)

[22] Bühler O, Holmes-Cerfon M. 2009 Particle dispersion by random waves in rotating shallow water. J. Fluid Mech. **638**, 5-26. (10.1017/S0022112009991091)

[23] Higgins C, Vanneste J. 2020 Lagrangian transport by deep-water surface gravity wavepackets: effects of directional spreading and stratification. J. Fluid Mech. **883**, A42. (10.1017/jfm.2019.877)

[24] Haney S, Young WR. 2017 Radiation of internal waves from groups of surface gravity waves. J. Fluid Mech. **829**, 280-303. (10.1017/jfm.2017.536)

[25] Frankel T. 2011 The geometry of physics. Cambridge, UK: Cambridge University Press.

[26] Holm DD. 2019 Stochastic closures for wave–current interaction dynamics. J. Nonlinear Sci. **29**, 2987-3031. (10.1007/s00332-019-09565-0) PMC7749098 33364683

[27] Holm DD. 2021 Stochastic variational formulations of fluid wave–current interaction. J. Nonlinear Sci. **31**, 4. (10.1007/s00332-020-09665-2) 33364683 PMC7749098

[28] Holm DD. 1996 The ideal Craik–Leibovich equations. Physica D **98**, 415-449. (10.1016/0167-2789(96)00105-4)

